# Mass Spectrometry Analysis of Vitreous Humor Reveals Novel Insights in Early and Late Onset Alzheimer's Disease

**DOI:** 10.1002/alz70856_107314

**Published:** 2026-01-09

**Authors:** Brittany T Ivey, Lauren Ball, Jennifer Bethard, Dariusz Pytel, Steven L Carroll

**Affiliations:** ^1^ Medical University of South Carolina, Charleston, SC, USA

## Abstract

**Background:**

More than 6 million Americans were living with Alzheimer's Disease (AD) in 2024, and that number is expected to triple over the next 40years. Although effective treatments for AD are becoming available, these are likely to be most effective when given early in the course of the disease. Unfortunately, biomarkers that effectively identify the early stages of AD are not yet available. However, there is growing interest in whether eye pathology can be used for this purpose. We have therefore performed mass spectrometry on 123 vitreous humor (VH) samples from autopsied patients with AD or AD‐related dementias (ADRDs) to identify potential biomarkers.

**Method:**

Following preparation with EasyPep 96 well plate kits, protein was reduced, alkylated, and digested with trypsin and Lys‐C. Resulting peptides were analyzed by LC‐MS/MS on an Orbitrap Exploris 480 MS in DIA mode. Pooled samples were analyzed in DDA mode to supplement database searching using Spectronaut 19.6 Direct‐DIA+. For protein identification, an FDR<0.01 and a minimum of 2 unique proteotypic peptides were required. Quantification was based on peak areas of 3‐6 MS2 fragment ions per peptide. Data were statistically evaluated using Perseus v1.6.15. Because of contamination with blood proteins, albumin and other blood proteins were removed. Median normalized, log2 transformed protein intensities were filtered to retain proteins quantified in 70% of the samples in one group and missing values were imputed from a down shifted normal distribution. Protein intensities were compared among groups using Student's t‐test. Log2 fold changes between groups, *p*‐values, and *q*‐values were reported.

**Result:**

Proteins identified through this approach have been implicated in neuronal development, neuroprotective functions, and neurodegenerative disorders. Proteins upregulated in early‐onset AD patients were found to be calcium modulators. Many proteins upregulated in late‐onset patients performed roles in neuroinflammation. Interestingly, a comparison of proteins between early and late onset AD revealed an increase in proteins involved in energy metabolism, splicing and ribosome assembly, and vesicular trafficking in early onset AD.

**Conclusion:**

The identification of novel AD biomarkers in the vitreous humor suggests that these proteomic analyses are identifying differences in the pathogenic mechanisms of early and late‐onset AD.

